# Rapamycin Mitigates Corneal Damage in a Mouse Model of Alkali Burn Injury

**DOI:** 10.3390/bioengineering12090998

**Published:** 2025-09-19

**Authors:** Basanta Bhujel, Woojune Hur, Seorin Lee, Hun Lee, Ho Seok Chung, Jae Yong Kim

**Affiliations:** 1 Department of Ophthalmology, Asan Medical Center, College of Medicine, University of Ulsan, Seoul 05505, Republic of Korea; basantabhujel86@gmail.com (B.B.); dnwnsgj@amc.seoul.kr (W.H.); ra02582@amc.seoul.kr (S.L.); yhun777@amc.seoul.kr (H.L.); 2 Department of Medical Science, Graduate School, University of Ulsan, Seoul 05505, Republic of Korea

**Keywords:** cornea, rapamycin, alkali burn, inflammation, neovascularization, fibrosis, ocular integrity

## Abstract

Alkali burns to the cornea cause severe damage characterized by an intense inflammatory response driven by inflammatory cytokines, which orchestrate pathological processes, including neovascularization, fibrosis, apoptosis, abnormal cell proliferation, and disorganization of the extracellular matrix (ECM), often resulting in permanent vision impairment or loss. Rapamycin (RAPA), a well-known mTOR inhibitor with potent immunosuppressive activity and pleiotropic therapeutic effects, was investigated as a novel restorative modality for promoting corneal wound healing in a mouse model of alkali burn injury. Topical RAPA treatment significantly reduced clinical signs of inflammation and decreased the infiltration of F4/80^+^ macrophages and CD45^+^ leukocytes, along with suppressed expression of pro-inflammatory cytokines (TNF-α, IL-1β, IL-6, and IL-17A). RAPA also markedly downregulated angiogenic mediators, such as VEGF, and endothelial markers, like CD31, resulting in significant inhibition of neovascularization. Furthermore, it prevented fibrotic tissue formation and myofibroblast activation, as evidenced by reduced α-SMA levels, and attenuated pathological matrix remodeling through decreased MMP-9 expression. Notably, RAPA preserved epithelial barrier function by maintaining the tight junction protein ZO-1 and reduced both apoptotic cell death (TUNEL) and dysregulated proliferation (Ki67^+^), thereby preserving the functional and structural integrity of the cornea. In conclusion, RAPA represents a promising therapeutic candidate for managing severe corneal alkali burn injuries, with the potential to enhance corneal wound healing, minimize long-term complications, and protect visual function.

## 1. Introduction

Alkali burns to the eye represent one of the most devastating forms of ocular surface injuries, often leading to severe and permanent visual impairment. The highly caustic nature of alkaline substances enables rapid penetration into corneal tissues, leading to widespread epithelial damage and the overall destruction of the corneal extracellular matrix (ECM) [1,2]. Following corneal alkali burn injury, a multifaceted pathological cascade is triggered, driven primarily by an intense inflammatory response that disrupts the tight epithelial barrier and exposes the underlying stromal tissues [3]. This damage prompts the immediate recruitment of innate immune cells, particularly neutrophils and macrophages, which migrate into the corneal stroma [4]. Neutrophils are among the earliest responders, releasing matrix metalloproteinases (MMPs) and pro-angiogenic factors, such as vascular endothelial growth factor (VEGF), thereby promoting ECM degradation and initiating neovascular sprouting [5,6]. This process contributes to a vicious cycle of degradation that exacerbates corneal tissue damage [7]. Likewise, leukocytes rapidly release abundant proteolytic enzymes and inflammatory mediators, further compromising ocular structures. Concurrently, macrophages infiltrate the stroma in large numbers and undergo polarization, with M1-type macrophages secreting pro-inflammatory cytokines including interleukin-1 beta (IL-1β), tumor necrosis factor-alpha (TNF-α), and interleukin-6 (IL-6), thereby intensifying the local inflammatory milieu [8,9]. These cytokines not only perpetuate inflammation but also enhance vascular permeability and endothelial cell proliferation, thereby accelerating pathological neovascularization [10].

In parallel, sustained inflammation leads to the activation and differentiation of fibroblasts into contractile myofibroblasts, characterized by the expression of alpha-smooth muscle actin (α-SMA), which contributes to excessive collagen deposition and pathological fibrotic remodeling of the corneal stroma [11]. Apoptosis of resident keratocytes and epithelial cells further undermines tissue integrity, hindering regenerative processes and promoting corneal opacity. Moreover, even mild corneal alkali burn injury may disrupt the ocular surface microenvironment, leading to altered corneal moisture content, destabilization of the tear film, and damage to adjacent supportive tissues, thereby compounding surface instability [12,13]. Thus, following the initial tissue injury, inflammation arises as a downstream response, culminating in vision-impairing scarring and delayed corneal wound healing. Taken together, this sequence of pathological events highlights the critical need for timely therapeutic intervention to preserve both corneal transparency and function [6,14,15].

Despite the severity of corneal alkali burn injury, current treatment options remain limited. Anti-inflammatory agents, such as corticosteroids and non-steroidal anti-inflammatory drugs (NSAIDs), are widely used but offer only partial relief and are associated with adverse effects, including increased intraocular pressure, corneal thinning, and delayed healing [16]. Anti-VEGF therapies effectively reduce neovascularization by targeting VEGF-A but fail to address the broader inflammatory processes involved in corneal alkali burn injury [17]. Biologics offer another therapeutic avenue; however, their high cost and potential for epithelial toxicity hinder widespread clinical use [18]. Importantly, these interventions primarily target isolated pathways and fail to engage the complex molecular and cellular processes required for complete corneal repair. Moreover, in severe cases, corneal transplantation is considered, but outcomes are often compromised by immune rejection and graft failure, especially in eyes with fibrovascular pannus formed post-injury [19]. Collectively, these limitations underscore a paramount unmet need for innovative strategies that not only suppress inflammation but also actively promote tissue regeneration and restore overall corneal integrity and function.

In this regard, rapamycin (RAPA), a well-characterized macrolide compound, was isolated in 1975 from *Streptomyces hygroscopicus*, a soil bacterium found on Easter Island [20]. Initially identified as an antifungal agent, RAPA later emerged as a powerful immunosuppressive compound, presenting a compelling therapeutic paradigm for managing complex inflammatory conditions [21]. RAPA functions as a potent inhibitor of the mammalian target of the rapamycin (mTOR) pathway, a crucial regulator of cellular processes including growth, proliferation, survival, and immune modulation [22]. Through mTOR inhibition, RAPA exerts broad immunosuppressive, anti-inflammatory, anti-fibrotic, and anti-proliferative effects [23,24,25,26]. Clinically, RAPA has been applied successfully to prevent organ transplant rejection and to treat various fibrotic and proliferative diseases [27,28]. Preclinical evidence further supports its capacity to attenuate inflammation and fibrosis in diverse tissues [21,23,29].

While previous studies have demonstrated that RAPA can reduce inflammation and neovascularization in corneal alkali burn injury, they primarily relied on systemic administration and focused on a limited range of pathological outcomes. A comprehensive evaluation of RAPA across the broad spectrum of injury-induced processes and functional outcomes in corneal wound healing remains lacking [23,24,25,26]. To bridge this gap, we systematically investigated the therapeutic potential of topical RAPA, a clinically relevant, patient-friendly route of administration. Given its pleiotropic actions, RAPA holds promise not only in dampening the central inflammatory and angiogenic cascades that drive corneal damage but also in fostering regenerative repair, which is crucial for corneal wound healing [29].

**Figure 1:**
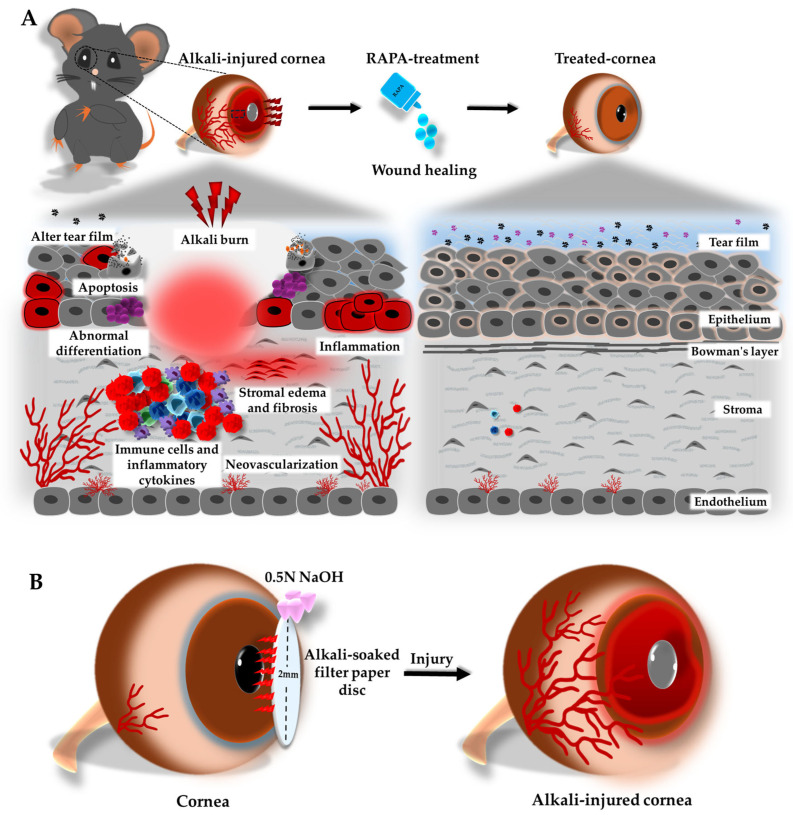
(**A**) Schematic illustration of the protective and reparative effects of RAPA in a mouse model of corneal alkali burn injury. The inflammatory response is a central driver of corneal damage, leading to robust immune cell infiltration and elevated pro-inflammatory cytokine activity. These events initiate a cascade of pathological changes, llcluding neovascularization, stromal fibrosis, edema, epithelial apoptosis, abnormal cell differentiation, and tear film instability. Topical RAPA treatment effectively attenuates these pathological changes, promoting corneal wound healing. (**B**) Diagrammatic representation of the corneal alkali burn injury method in a mouse, where a 2-mm alkali-soaked filter paper disc is applied to the corneal surface to induce injury and trigger a wave of pathological responses.

Investigating topical RAPA as a pleiotropic interventional candidate, we aimed to pave the way for next-generation therapeutic horizons that holistically address both injury resolution and tissue restoration, providing a strong scientific rationale for its clinical exploration and opening new avenues to enhance corneal wound healing (Figure 1A). Collectively, these findings may provide fundamental insights into novel treatment frontiers for managing severe corneal alkali burn injuries and improving visual outcomes in affected patients.

## 2. Materials and Methods

### 2.1. Animals

A total of twenty-seven female C57BL/6 mice (8 weeks old, 20–25 g; Orient Bio, Seongnam, Republic of Korea) were housed under standardized laboratory conditions, including a 12-h light/dark cycle and ad libitum access to sterilized food and water. All mice were allowed to acclimate for one week before the start of the experiment.

### 2.2. Ethics Statement

All animal experimental procedures were approved by the Animal Ethics Committee of the University of Ulsan College of Medicine and conducted in full compliance with the guidelines established by the Institutional Animal Care and Use Committee (IACUC) of the University of Ulsan (IACUC A20242479).

### 2.3. Mouse Model of Corneal Alkali Burn Injury and Experimental Groups

The mouse model of corneal alkali burn injury was established according to previously reported protocols [1,30]. Briefly, mice were anesthetized via intraperitoneal injection of Zoletil^®^ (50 mg/kg; Virbac Laboratories, Carros, France) and Rompun^®^ (10 mg/kg; Bayer, Seoul, Republic of Korea). A 2-mm filter paper disc, presoaked in 0.5 N sodium hydroxide (NaOH), was gently applied to the center of the cornea for 10 s to induce alkali burn injury. The disc was then promptly removed, and the eye was thoroughly irrigated with sterile PBS for 1 min to wash out any residual alkali (Figure 1B).

On the first day following alkali burn injury, all mice were randomly allocated into three groups: (1) normal (untreated, no injury), (2) injury-only, and (3) injury with rapamycin treatment (injury + RAPA) (*n* = 9/each group). The injury + RAPA-treatment group received topically RAPA (1 mg/mL; Sigma-Aldrich, St. Louis, MO, USA), administered three times daily for 14 consecutive days. The RAPA concentration employed in this study was based on a previously published report [29]. On day 15, all animals were humanely euthanized by CO_2_ asphyxiation, and the corneas were collected for subsequent histological, protein, and immunohistochemical analyses.

### 2.4. Evaluation of Corneal Clinical Score

The corneal clinical score was evaluated based on the previously established method [31]. Briefly, corneal clarity was graded on a scale from 0 to 4: 0, a completely clear cornea; 1, mild haze with the pupil still visible; 2, moderate opacity with the pupil partially visible; 3, dense opacity making the pupil barely discernible; and 4, total opacity with the pupil no longer visible.

### 2.5. Tear Secretion Measurements

**Figure 2:**
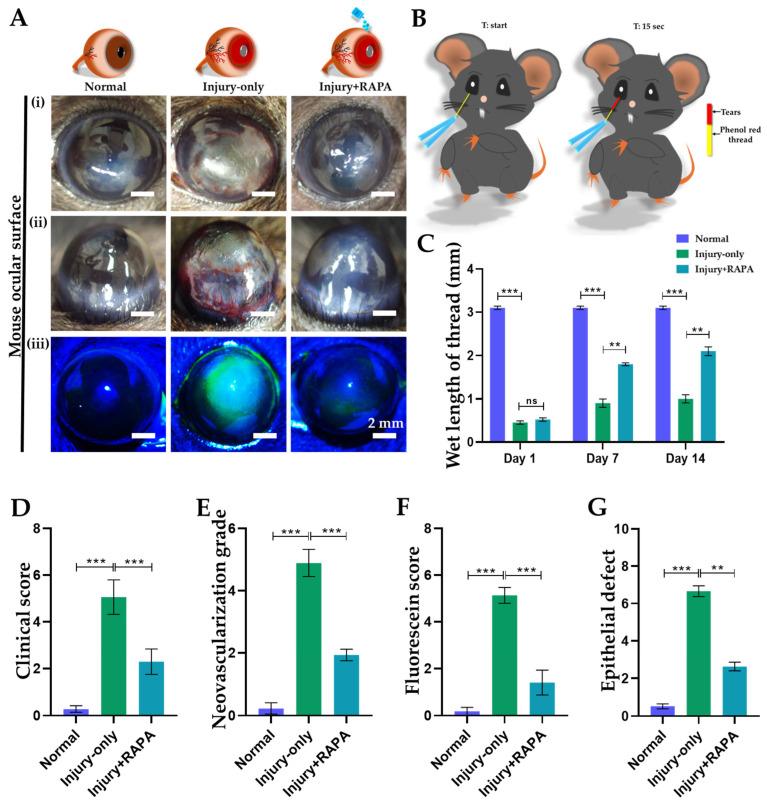
Effects of RAPA on the ocular surface in a mouse model of corneal alkali burn injury. (**A**): (**i**,**ii**) Slit lamp examination of corneas in the normal, injury-only, and injury + RAPA-treated groups. (**iii**) Fluorescein staining of corneas in the normal, injury-only, and injury + RAPA-treated groups. (**B**) Illustration of the phenol red thread test for evaluating tear secretion in mice, showing the thread positioned at the start time (t-start) and left on the eye for 15 s (t:15 s) before measuring tear absorption length in millimeters (mm). (**C**) Tear-secretion rate (measured in mm wetted within 15 s) in the normal, injury-only, and injury + RAPA-treated groups on days 1, 7, and 14. (**D**) Changes in corneal clinical scores in the normal, injury-only, and injury + RAPA-treated groups. (**E**) Changes in corneal neovascularization grade in the normal, injury-only, and injury + RAPA-treated groups. (**F**) Changes in corneal fluorescein staining scores in the normal, injury-only, and injury + RAPA-treated groups. (**G**) Changes in corneal epithelial defect in the normal, injury-only, and injury + RAPA-treated groups. In (**C**–**G**), data represent mean ± SEM (*n* = 9). One-way ANOVA followed by the Tukey test. * *p* < 0.05, ** *p* < 0.01, *** *p* < 0.001, ns, not significant.

Tear secretion was quantified using the Zone-Quick phenol red thread test following a previously established protocol [32]. The phenol red thread was positioned at the lateral aspect of the lower conjunctival fornix for 15 s. Upon contact with tear fluid, the yellow thread rapidly turned red, and the moistened segment was promptly recorded using a digital caliper (Monos, Seoul, Republic of Korea) (Figure 2B). Measurements were conducted on days 1, 7, and 14 following induction of corneal alkali burn injury to monitor changes in tear production over time.

### 2.6. Corneal Fluorescein Staining and Scoring

Corneal epithelial injury was assessed by applying 1% fluorescein sodium solution (Sigma-Aldrich, Darmstadt, Germany) directly onto the corneal surface. After 2 min, the excess dye was flushed away with artificial tears to minimize nonspecific staining. Corneal fluorescence was visualized under cobalt blue illumination using a microscope in a darkened environment. Images were captured, and the extent of corneal epithelial injury was quantified using ImageJ software (version 1.62f).

The severity of fluorescein staining was measured on a scale of 0 to 4, according to the percentage of the corneal surface affected [33]. Scoring was performed in a blinded manner by three independent observers. The grading criteria were as follows: grade 0, no staining; 0.5, mild punctate staining; 1, widespread punctate staining; 2, diffuse staining involving less than one-third of the corneal surface; 3, diffuse staining spanning more than one-third of the cornea; and 4, diffuse staining extending across more than two-thirds of the cornea.

### 2.7. Corneal Neovascularization Assessment

Corneal neovascularization was assessed using a previously established method [34]. The grading criteria for corneal neovascularization were as follows: grade 0, no neovascularization, with no new vessels from the limbus; grade 1, mild neovascularization, with new vessels originating from the limbus; grade 2, moderate neovascularization, where blood vessels extended from the limbus toward the center of the cornea; and grade 3, severe neovascularization, with vessels reaching or crossing the central cornea.

### 2.8. Histological Analysis

Tissue samples were first embedded in paraffin and then cut into 5-µm-thick sections using a microtome (Leica, Wetzlar, Germany). The sections were dewaxed, rehydrated, and stained with hematoxylin and eosin (H&E; Abcam, Cambridge, UK) and Masson’s Trichrome (MT; Sigma, St. Louis, MO, USA) according to established protocols. The sections were cleared in xylene, mounted, and examined under a microscope.

### 2.9. Immunofluorescence Staining

For immunostaining, 5-µm paraffin-embedded sections were deparaffinized, rehydrated, and stained with primary antibodies targeting: ZO-1 (ab617300; Abcam, 1:200), F4/80 (ab6640; Abcam, 1:200), CD45 (ab23910; Abcam, 1:200), IL-1β (ab315084; Abcam, 1:100), VEGF (MA1-16629; Invitrogen, Waltham, MA, USA, 1:100), CD31 (ab230718; Abcam, 1:100), α-SMA (ab5694; Abcam, 1:100), MMP-9 (ab73734; Abcam,1:200), and Ki67 (ab15580; Abcam, 1:200). After 24 h of incubation, sections were washed with PBS and subsequently incubated in the dark at room temperature for 1 h with secondary antibodies conjugated to Alexa Fluor 488 (A11008; Invitrogen, 1:400), Alexa Fluor 555 (A21424; Invitrogen, 1:400), Alexa Fluor 568 (A10042; Invitrogen, 1:400), Alexa Fluor 647 (A21469; Invitrogen, 1:400), or Alexa Fluor 488 (A11029; Invitrogen, 1:400). Nuclei were counterstained with DAPI for 10 min, after which the sections were mounted using antifade medium. Images were acquired using a confocal microscope (Carl Zeiss, Jena, Germany), and fluorescence intensities were quantified using ImageJ software (version 1.62f).

### 2.10. Western Blot

Following euthanasia, corneal tissues were harvested, homogenized, and lysed using RIPA buffer supplemented with protease inhibitors. Proteins of equal concentration were separated by SDS-PAGE, transferred to PVDF membranes, and probed with primary antibodies against TNF-α (3707; Cell Signaling Technology, Danvers, MA, USA, 1:1000), IL-6 (ab9324; Abcam, 1:1000), IL-17A (ab79056; Abcam, 1:1000), and GAPDH (2118S; Cell Signaling Technology, 1:10,000). Following washing, membranes were treated with HRP-conjugated secondary antibodies. Protein bands were detected using a chemiluminescence detection system (WBKLS0100; MilliporeSigma, Burlington, VT, USA).

### 2.11. TUNEL Assay

Corneal tissues were fixed in paraffin and sectioned into 5-µm slices using a Leica microtome (Wetzlar, Germany). After dewaxing and rehydration, TUNEL staining (Roche, Munich, Germany; 684817910) was performed to detect apoptotic cells. Nuclei were counterstained, and imaging was performed with a Carl Zeiss confocal microscope (Jena, Germany).

### 2.12. Statistical Analysis

All data were expressed as mean ± the standard error of the mean (SEM). Statistical analyses were performed using GraphPad Prism 5.01 (GraphPad Software, Boston, MA, USA), and image quantification was conducted with ImageJ (version 1.62f). Group comparisons were analyzed by one-way ANOVA followed by Tukey’s test. Bartlett’s test was used to assess variance in the in vivo experiments. Statistical significance was denoted by a *p*-value < 0.05.

## 3. Results

### 3.1. RAPA Preserves Corneal Integrity in a Mouse Model of Corneal Alkali Burn Injury

The effect of RAPA on ocular lesions was assessed by measuring fluorescein staining scores and epithelial defect areas in the normal, injury-only, and injury + RAPA-treated groups in our mouse model of corneal alkali burn injury (Figure 2A(iii)).

Corneal fluorescein staining scores were significantly reduced in the injury + RAPA-treated group compared to the injury-only group, indicating that RAPA treatment preserves corneal epithelial integrity following alkali burn injury. In contrast, the normal group showed no staining (Figure 2F).

Consistently, the extent of epithelial defects was notably greater in the injury-only group, as evidenced by widespread fluorescein uptake and irregular staining patterns. In contrast, RAPA-treated corneas exhibited smaller and more localized staining areas, indicating reduced epithelial damage and accelerated wound closure in response to treatment (Figure 2G).

Together, these results suggest that RAPA effectively preserves corneal structure and promotes epithelial healing and clarity following alkali burn injury.

### 3.2. RAPA Reduces Corneal Clinical Scores in a Mouse Model of Corneal Alkali Burn Injury

We further assessed corneal inflammation by evaluating the clinical score. Interestingly, the score was substantially raised in the injury-only group, revealing pronounced inflammatory changes following alkali burn injury. Prominently, treatment with RAPA (injury + RAPA) markedly reduced the clinical score compared to the injury-only group (Figure 2A(i),D), suggesting that RAPA effectively alleviates corneal inflammation and promotes ocular surface recovery.

### 3.3. RAPA Attenuates Corneal Neovascularization Grade in a Mouse Model of Corneal Alkali Burn Injury

We further performed corneal neovascularization grading in our mouse model to assess pathological neovascularization. In our study, the injury-only group exhibited a dramatically elevated neovascularization grade compared to the normal group, reflecting extensive pathological angiogenesis following alkali burn injury. Notably, RAPA treatment (injury + RAPA) significantly attenuated the neovascularization grade relative to the injury-only group, underscoring its potential in suppressing abnormal vascular growth and supporting corneal wound healing (Figure 2A(ii),E). Consistent with our findings, previous studies have reported that corneal alkali burn injury induces robust and sustained corneal neovascularization, which contributes to impaired vision and delayed wound healing [35,36,37,38,39].

### 3.4. RAPA Enhances Tear Production in a Mouse Model of Corneal Alkali Burn Injury

Next, tear production was measured in all groups using the phenol red thread test. A previous study has already demonstrated that corneal alkali burn injury disrupts tear secretion in an animal model [40]. Consistent with this finding, the injury-only group exhibited a significant decrease in tear production, indicating impaired lacrimal function following alkali-induced ocular surface damage. Importantly, treatment with RAPA (injury + RAPA) significantly enhanced tear production compared to the injury-only group, demonstrating its potential to restore tear secretion and maintain ocular surface homeostasis following alkali burn injury (Figure 2B,C).

### 3.5. RAPA Restores Corneal Epithelial Barrier Function by Maintaining ZO-1 Expression in a Mouse Model of Corneal Alkali Burn Injury

Disruption of the corneal epithelial barrier is a pivotal aspect of corneal alkali burn injury, mainly due to the breakdown of tight junction proteins such as zonula occludens-1 (ZO-1), which are vital for maintaining epithelial cell-cell adhesion and barrier function [41,42].

**Figure 3:**
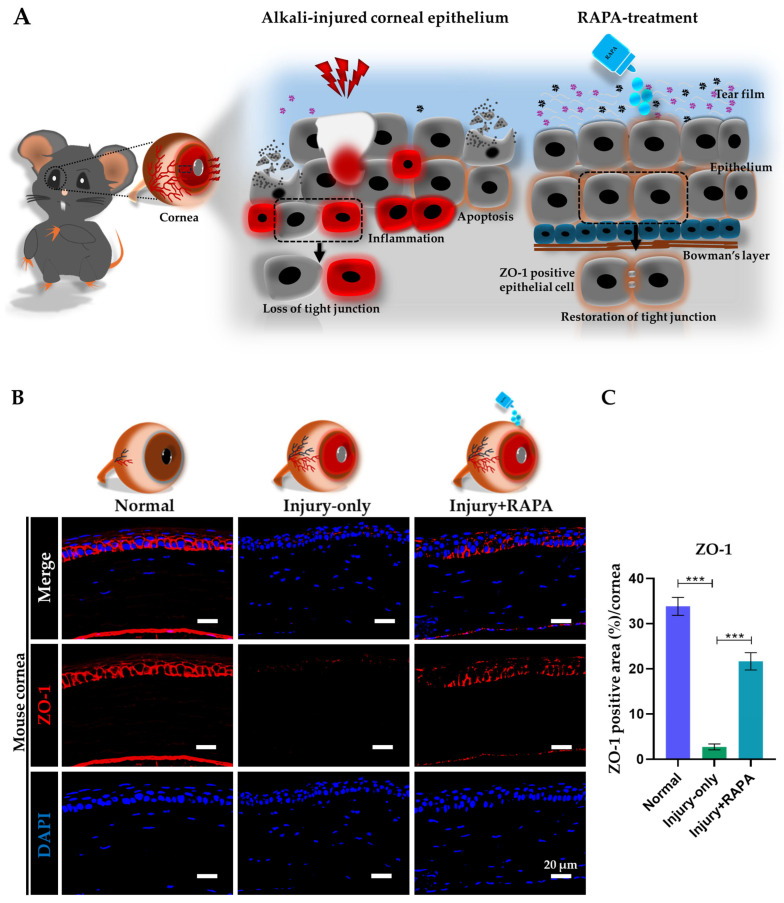
Effects of RAPA on corneal epithelial barrier function in a mouse model of corneal alkali burn injury. (**A**) Schematic representation of the disintegration of ZO-1 tight junctions following corneal alkali burn injury. The injury compromises corneal epithelial barrier function, resulting in the loss of ZO-1 expression and disruption of epithelial integrity. RAPA treatment restores ZO-1 expression, indicating recovery of corneal epithelial function, maintenance of epithelial integrity, and reestablishment of tight barrier function. (**B**) Immunofluorescence staining for ZO-1 in the corneal epithelium of the normal, injury-only, and injury + RAPA-treated groups. (**C**) Changes in the expression of ZO-1. In (**C**), data represent mean ± SEM (*n* = 6). One-way ANOVA followed by the Tukey test. * *p* < 0.05, ** *p* < 0.01, *** *p* < 0.001.

Additionally, we studied the role of RAPA in modulating ZO-1 expression in a mouse model of corneal alkali burn injury. Immunofluorescence staining revealed that, in untreated injured corneal epithelium, ZO-1 expression was severely disrupted, showing fragmented and diffuse patterns compared to the continuous, belt-like distribution observed in normal corneas. By contrast, RAPA-treated corneas exhibited a marked restoration of ZO-1 localization at epithelial cell junctions (Figure 3B,C), closely resembling the intact tight junction pattern seen in normal corneas.

### 3.6. RAPA Reduces Fibrotic Tissue Deposition and Inflammatory Cell Infiltration in the Corneal Stroma of a Mouse Model of Corneal Alkali Burn Injury

**Figure 4:**
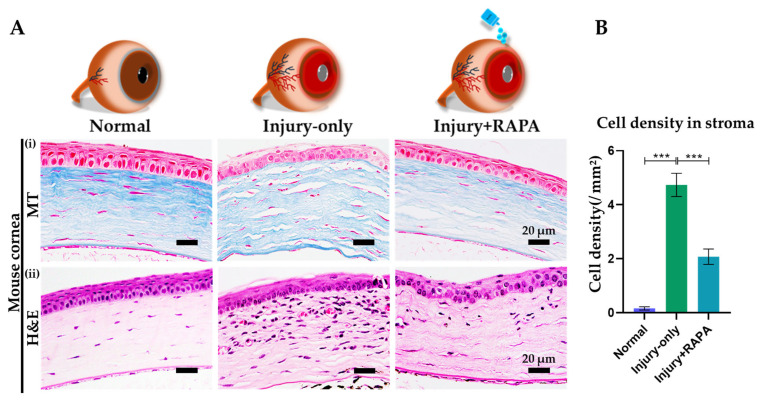
Effect of RAPA on the fibrotic alterations and inflammatory cell infiltration in the corneal stroma in the mouse model of corneal alkali burn injury. (**A**): (**i**) MT staining of mouse corneas to assess fibrotic tissue alteration in the normal, injury-only, and injury + RAPA-treated groups. (**ii**) H&E staining to evaluate inflammatory cell infiltration in the corneal stroma. (**B**) Changes in stromal inflammatory cell density among the groups. In (**B**), data represent mean ± SEM (*n* = 6). One-way ANOVA followed by the Tukey test. * *p* < 0.05, ** *p* < 0.01, *** *p* < 0.001.

We performed MT staining to investigate fibrotic alterations in the corneal stroma in our mouse model. The injury-only group showed widespread fibrosis, scarring, and impaired healing characterized by a loss of the normal parallel lamellar arrangement and a disorganized ECM structure, mirroring the structural abnormalities described in earlier corneal alkali burn models [36,43,44]. Surprisingly, treatment with RAPA significantly alleviated fibrotic tissue accumulation, resembling that of the normal cornea (Figure 4A(i)).

Further, H&E staining was carried out to assess inflammatory cell infiltration in the corneal stroma in our mouse model. Strikingly, the injury-only group demonstrated a prominent density of inflammatory cells throughout the corneal stroma. This robust cellular infiltration reflects acute inflammation and aligns with the immune response typically observed following corneal alkali burn injury, as reported in previous studies [9,24,45,46]. In contrast, RAPA treatment substantially reduced inflammatory cell infiltration (Figure 4A(ii),B). These observations indicate that RAPA effectively mitigates the inflammatory response, thereby fostering a more conducive environment for corneal wound healing and tissue repair.

### 3.7. RAPA Diminishes Macrophage and Pan-Leukocyte Infiltration in the Cornea of a Mouse Model of Corneal Alkali Burn Injury

Inflammation triggered by immune cell infiltration plays a central role in exacerbating corneal tissue damage and delaying repair following corneal alkali burn injury. Macrophages serve as key mediators of this inflammatory response, orchestrating tissue remodeling and immune activation [47]. At the same time, infiltration of pan-leukocytes contributes to the overall inflammatory milieu and affects healing outcomes [48].

**Figure 5:**
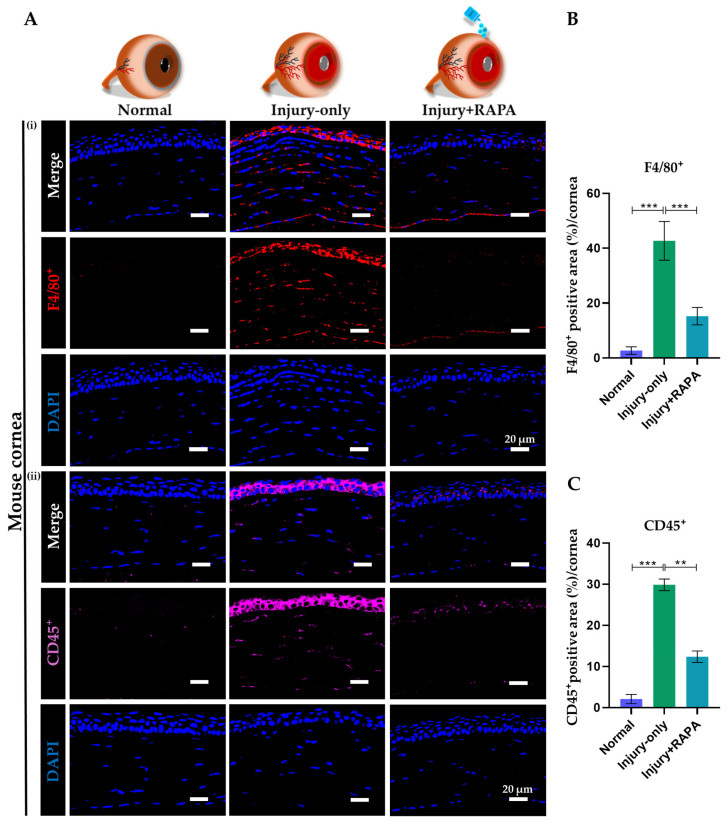
Effects of RAPA on macrophage and pan-leukocyte infiltration in the cornea of a mouse model of corneal alkali burn injury. (**A**) Immunofluorescence staining for (**i**) F4/80^+^ and (**ii**) CD45^+^ in the corneas of the normal, injury-only, and injury + RAPA-treated groups. Changes in the expression of (**B**) F4/80^+^ and (**C**) CD45^+^. In (**B**,**C**), data represent mean ± SEM (*n* = 6). One-way ANOVA followed by the Tukey test. * *p* < 0.05, ** *p* < 0.01, *** *p* < 0.001.

To assess immune cell infiltration following corneal alkali burn injury, immunofluorescence staining for F4/80^+^ (macrophages) and CD45^+^ (pan-leukocytes) was performed. The injury-only group exhibited a marked increase in both F4/80^+^ macrophages and CD45^+^ leukocytes compared to the normal group, indicating exacerbated inflammatory cell recruitment due to the injury. Conversely, treatment with RAPA pronouncedly reduced the number of both F4/80^+^ and CD45^+^ cells, suggesting that RAPA effectively suppresses macrophage and pan-leukocyte infiltration, thereby exerting a strong anti-inflammatory effect on the injured cornea (Figure 5A–C).

### 3.8. RAPA Downregulates Pro-Inflammatory Cytokines in the Cornea of a Mouse Model of Corneal Alkali Burn Injury

Subsequently, we assessed the corneal pro-inflammatory response following corneal alkali burn injury by immunofluorescence staining for IL-1β and western blotting for TNF-α, IL-6, and IL-17A. These cytokines play an imperative role in mediating the acute inflammatory response and are well-established as reliable markers of inflammation and tissue damage in corneal alkali burn injury models [39,49].

**Figure 6:**
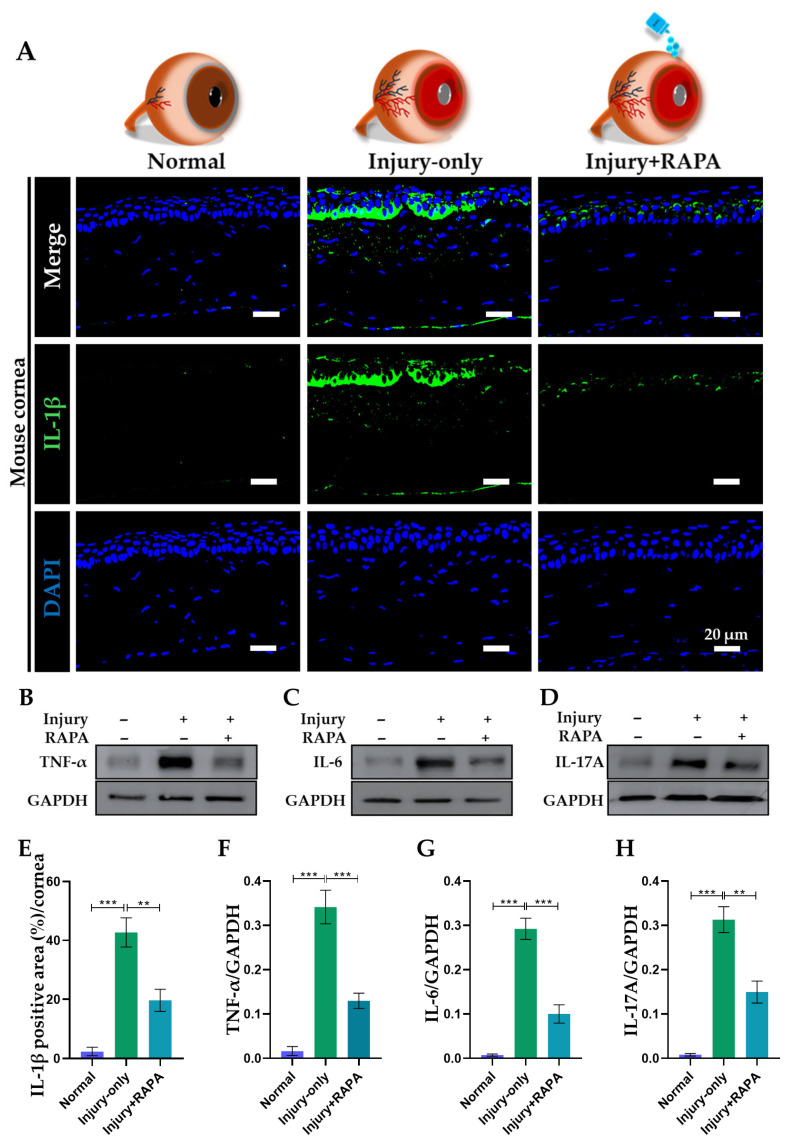
Effects of RAPA on pro-inflammatory cytokines in the cornea of a mouse model of corneal alkali burn injury. (**A**) Immunofluorescence staining for IL-1β in the corneas of the normal, injury-only, and injury + RAPA-treated groups. Western blot analysis of (**B**) TNF-α, (**C**) IL-6, and (**D**) IL-17A. (**E**) Changes in the expression of IL-1β. Relative expression of (**F**) TNF-α, (**G**) IL-6, and (**H**) IL-17A normalized to GAPDH. In (**E**–**H**), data represent mean ± SEM (*n* = 6, *n* = 3). One-way ANOVA followed by the Tukey test. * *p* < 0.05, ** *p* < 0.01, *** *p* < 0.001.

In the injury group, we observed robust immunoreactivity for IL-1β and a marked increase in the protein expression levels of TNF-α, IL-6, and IL-17A, confirming a strong inflammatory response that parallels earlier research in corneal alkali burn injury [9,38]. In contrast, corneas from RAPA-treated mice showed considerably reduced expression of IL-1β (Figure 6A,E) and lower protein levels of TNF-α, IL-6, and IL-17A, as demonstrated by western blot analysis (Figure 6B–D,F–H).

These findings underscore the potent anti-inflammatory effect of RAPA, which effectively downregulates key cytokines, thereby limiting excessive inflammation and fostering a more favorable environment for tissue repair following corneal alkali burn injury.

### 3.9. RAPA Represses Angiogenic Mediator and Endothelial Marker in the Cornea of a Mouse Model of Corneal Alkali Burn Injury

We then performed immunofluorescence staining for VEGF and CD31^+^ in corneal tissue to demonstrate angiogenesis and endothelial marker, respectively, in our mouse model of corneal alkali burn injury. Pathological angiogenesis is a common and detrimental consequence of corneal alkali burn injury, contributing to inflammation, scarring, and vision loss [14,36,50].

**Figure 7:**
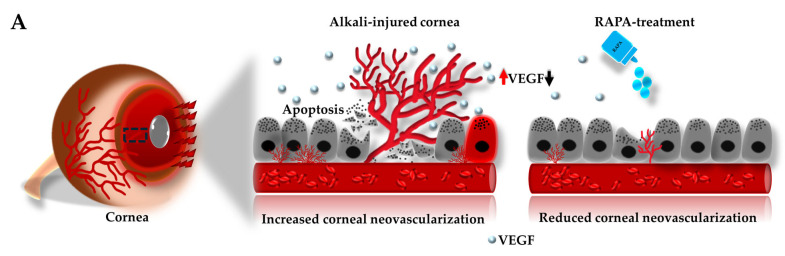

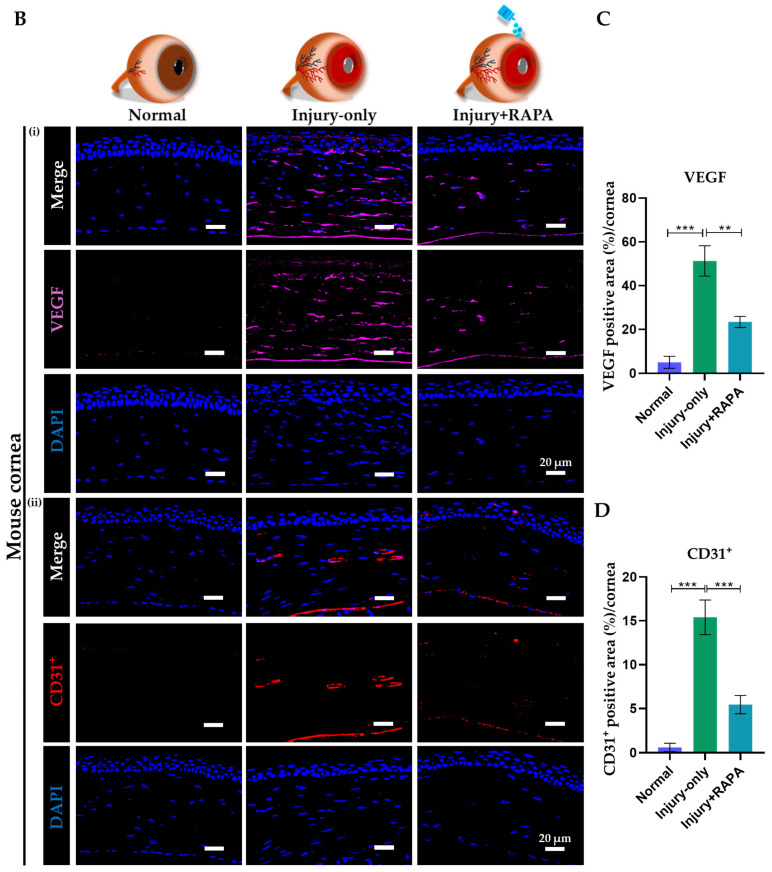
Effects of RAPA on angiogenic mediators and endothelial markers in the cornea of a mouse model of corneal alkali burn injury. (**A**) Schematic representation of increased VEGF expression in the cornea following alkali burn injury, which promotes pathological neovascularization. RAPA treatment downregulated VEGF expression, resulting in reduced neovascularization in the cornea. (**B**) Immunofluorescence staining for (**i**)VEGF and (**ii**) CD31^+^ in the corneas of the normal, injury-only, and injury + RAPA-treated groups. Changes in the expression of (**C**) VEGF and (**D**) CD31^+^. In (**C**,**D**), data represent mean ± SEM (*n* = 6). One-way ANOVA followed by the Tukey test. * *p* < 0.05, ** *p* < 0.01, *** *p* < 0.001.

Our data revealed heightened VEGF and CD31^+^ expression levels in the corneal stroma of the injury-only group compared to the normal, underscoring intensified angiogenic activity. However, treatment with RAPA markedly reduced VEGF expression (Figure 7B–D), suggesting inhibition of pathological neovascularization and protection of the avascular status of the cornea, essential for successful corneal wound healing.

### 3.10. RAPA Suppresses α-SMA–Positive Myofibroblast-Associated Fibrosis and MMP-9–Mediated Pathological Matrix Remodeling in the Cornea of a Mouse Model of Corneal Alkali Burn Injury

Additionally, immunofluorescence staining was performed to detect α-SMA, a prominent indicator of myofibroblasts that play a central role in fibrotic tissue remodeling, and MMP-9, a key MMP involved in ECM degradation and pathological tissue remodeling in the cornea. As indicated by previous studies, excessive myofibroblast activation and upregulation of MMP-9 contribute to pathological fibrosis and impaired healing following corneal alkali burn injury [11,51].

**Figure 8:**
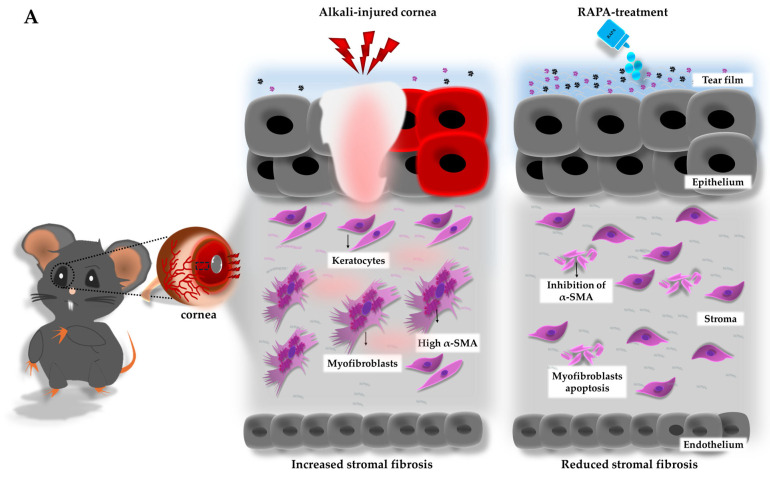

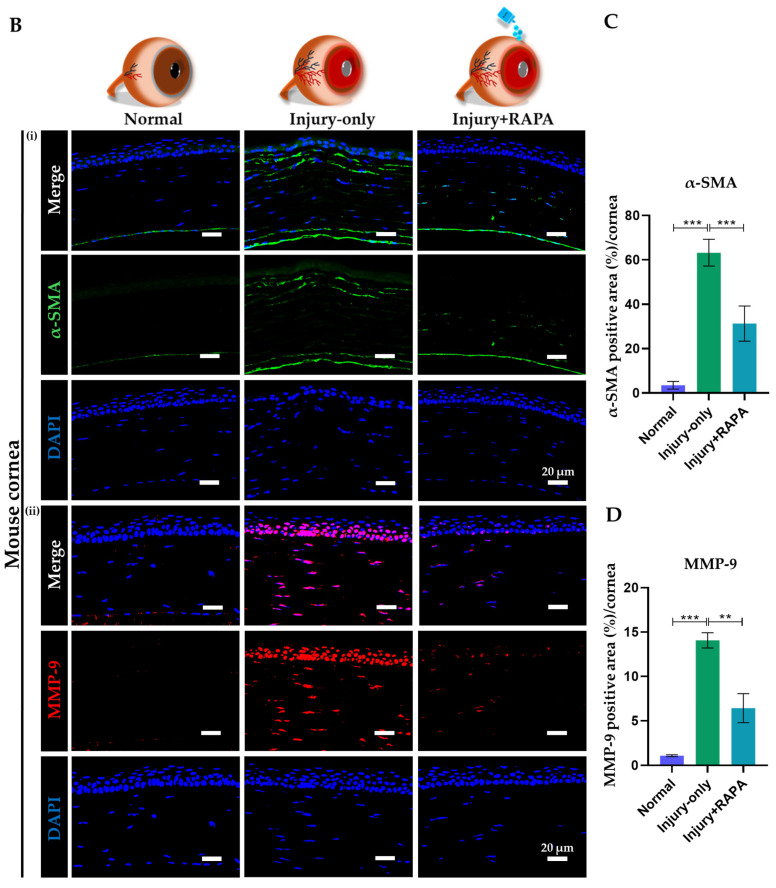
Effects of RAPA on α-SMA–positive myofibroblast-associated fibrosis and MMP-9-mediated pathological matrix remodeling. (**A**) Schematic representation of fibrotic tissue remodeling leading to corneal scarring following alkali burn injury. The injury induces the transformation of resident corneal fibroblasts into contractile myofibroblasts expressing α-SMA, resulting in excessive ECM deposition and stromal disorganization, which contributes to corneal haze and visual impairment. In contrast, RAPA treatment inhibits α-SMA expression, suppresses myofibroblast differentiation, and induces myofibroblast apoptosis, thereby reducing fibrosis and preserving corneal transparency. (**B**) Immunofluorescence staining for (**i**) α-SMA and (**ii**) MMP-9 in the corneas of the normal, injury-only, and injury + RAPA-treated groups. Changes in the expression of (**C**) α-SMA and (**D**) MMP-9. In (**C**,**D**), data represent mean ± SEM (*n* = 6). One-way ANOVA followed by the Tukey test. * *p* < 0.05, ** *p* < 0.01, *** *p* < 0.001.

Our results revealed that alkali burn injury noticeably increased α-SMA expression in the corneal stroma, signifying active myofibroblast-mediated fibrosis. Concurrently, MMP-9 was detected in both the corneal epithelium and stroma, reflecting excessive ECM degradation and aberrant tissue remodeling. Importantly, RAPA treatment prominently reduced the expression of both α-SMA and MMP-9, demonstrating its strong anti-fibrotic effect and its ability to suppress pathological matrix remodeling (Figure 8B–D).

These findings underscore RAPA’s clinical value in promoting balanced tissue repair and preventing fibrotic scarring in the cornea following alkali burn injury.

### 3.11. RAPA Inhibits Cellular Apoptosis in the Cornea of a Mouse Model of Corneal Alkali Burn Injury

The corneal epithelium is highly vulnerable to chemical insults, such as alkali burns, which can rapidly induce apoptosis and compromise tissue integrity [52]. To evaluate cell death following alkali injury, TUNEL staining was performed on the corneal tissue.

**Figure 9:**
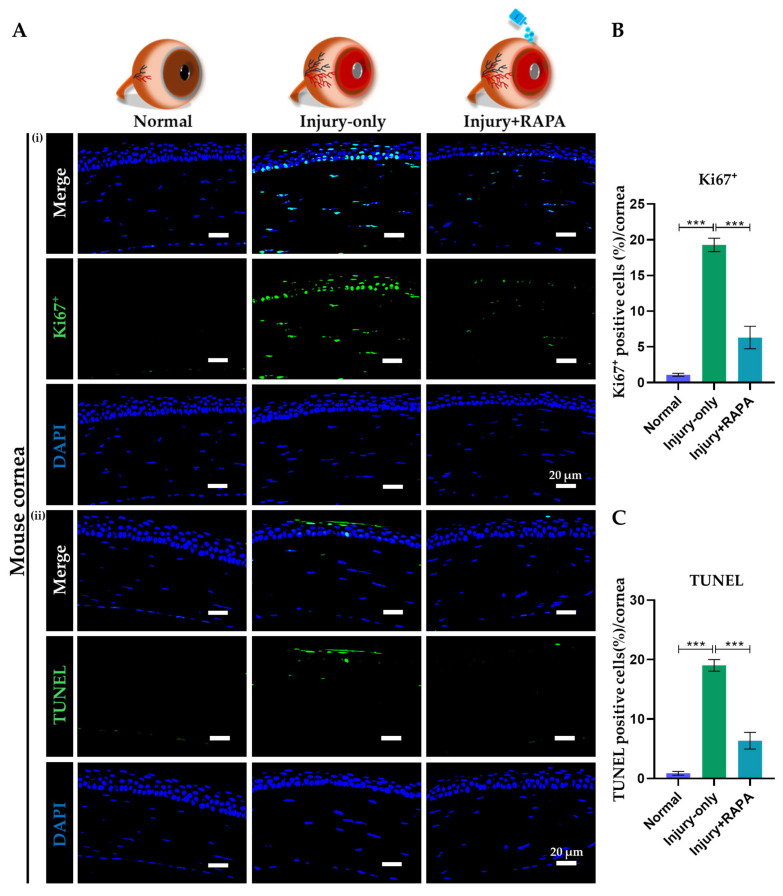
Effects of RAPA on dysregulated cell proliferation and cellular apoptosis in the cornea of a mouse model of corneal alkali burn injury. (**A**) Immunofluorescence staining for (**i**) Ki67^+^ and (**ii**) TUNEL in the corneas of the normal, injury-only, and injury + RAPA-treated groups. Changes in the expression of (**B**) Ki67^+^ and (**C**) TUNEL-positive cells. In (**B**,**C**), data represent mean ± SEM (*n* = 6). One-way ANOVA followed by the Tukey test. * *p* < 0.05, ** *p* < 0.01, *** *p* < 0.001.

In our study, the injury-only group exhibited a high number of apoptotic cells on the apical layer of the corneal epithelium, substantiating the presence of vast apoptotic activity. Clearly, the injury + RAPA-treated group showed a marked reduction in apoptotic cells compared to the untreated group (Figure 9A(i),B), suggesting that RAPA confers a protective effect by attenuating apoptosis and promoting corneal cell survival and enhancing regenerative capability following corneal alkali burn injury.

### 3.12. RAPA Limits Uncontrolled Cell Proliferation in the Cornea of a Mouse Model of Corneal Alkali Burn Injury

To assess dysregulated proliferative activity, Ki67^+^ immunostaining was performed on corneal tissue. Our results revealed a substantial elevation in Ki67^+^ cells, predominantly localized within the basal epithelial layer of the injury-only group, reflecting heightened pathological proliferation in response to corneal alkali burn injury.

Interestingly, the injury + RAPA-treated group showed a marked reduction in Ki67^+^ cells compared to the injury-only group (Figure 9B(ii),C), suggesting that RAPA effectively suppresses aberrant proliferation and promotes the restoration of epithelial homeostasis.

## 4. Discussion

Corneal alkali burn injury is among the most sight-threatening ocular injuries, primarily because the initial trauma triggers a profound inflammatory cascade that leads to progressive corneal deterioration. This intense inflammation contributes to neovascularization, ulceration, severe keratitis, and scarring, often resulting in significant vision loss or even permanent blindness if not promptly managed [53,54,55]. As an ophthalmic emergency, corneal alkali burn injury requires immediate intervention to halt the progression of corneal damage and preserve visual function. Although this has been well-documented, current therapies remain insufficient to effectively support corneal wound healing and fully restore both structural and functional integrity [56].

In this study, we demonstrated that topical RAPA, an mTOR inhibitor with well-established immunoregulatory and tissue-protective properties, exerts pronounced curative efficacy in a mouse model of corneal alkali burn injury [24,25,29,55]. The key findings of our study are as follows: RAPA treatment (1) restored ocular surface integrity, (2) reestablished corneal epithelial barrier function, (3) suppressed the expression of pro-inflammatory cytokines, (4) attenuated corneal neovascularization, (5) reduced macrophage and leukocyte infiltration, (6) downregulated angiogenic mediators and endothelial markers, (7) inhibited myofibroblast-associated fibrosis and pathological matrix remodeling, (8) curtailed excessive dysregulated cell proliferation and mitigated cellular apoptosis, and (9) enhanced tear production.

Corneal alkali burn injury elicits a robust inflammatory response, involving a multitude of cells and aberrant growth factors that severely disrupt tissue repair and regeneration. The damaged epithelial and stromal cells, together with infiltrating leukocytes, rapidly release a range of cytokines with overlapping pro-inflammatory, pro-angiogenic, and pro-fibrotic effects, activating multiple cell signaling pathways [54]. This response further promotes the influx of neutrophils and macrophages, amplifying the local inflammatory environment. Subsequently, levels of key pro-inflammatory mediators, such as TNF-α, IL-1β, IL-6, IL-17A, toll-like receptor 4 (TLR4), NOD-like receptor family pyrin domain-containing 3 (NLRP3), and caspase 1, are markedly elevated. These mediators jointly exacerbate corneal tissue damage and delay repair, as observed in preclinical models of corneal alkali burn injury, outlining the complex and challenging landscape of inflammatory mediators involved [24,25,57,58,59,60].

In alignment with previous findings, our study demonstrated that RAPA treatment profoundly suppressed corneal inflammation [24]. Specifically, we observed a significant reduction in F4/80^+^ macrophage infiltration, decreased CD45^+^ pan-leukocyte accumulation, and downregulated expression of pro-inflammatory cytokines, including TNF-α, IL-1β, IL-6, and IL-17A, in RAPA-treated corneas. Consistent with previous reports, IL-1β and CD45^+^ cells were predominantly localized within the corneal epithelium, underscoring this layer as a critical hub of inflammatory activation following corneal alkali burn injury [61,62]. Our findings further corroborate earlier reports indicating that the inflammatory response reaches its peak within the first two weeks post-injury [58,63].

Taken together, these findings highlight RAPA’s potent anti-inflammatory properties and its capacity to attenuate waves of acute immune activation and subsequently restrain pro-inflammatory cytokine expression following corneal alkali burn injury, thereby supporting its therapeutic value for preserving corneal integrity and promoting effective wound healing.

Crucially, neovascularization is a pivotal pathological sequela of corneal alkali burn injury, wherein disruption of the avascular corneal environment undermines both transparency and immune privilege [64]. This process is closely associated with inflammation, where tissue hypoxia and infiltrating immune cells (predominantly macrophages and neutrophils) drive the upregulation of pro-angiogenic mediators such as VEGF-A, thereby facilitating endothelial cell proliferation, migration, and new vessel formation [16,65,66,67,68]. This VEGF-driven signaling promotes pathological angiogenesis, while the presence of CD31^+^ endothelial cells reflects augmented vascular density and active neovascular remodeling in the cornea [69,70,71]. The mechanistic relationship between VEGF and CD31^+^ underlies the progression of neovascularization, where boosted VEGF levels enhance endothelial activation and subsequent CD31^+^ expression, as observed in the mouse model of corneal neovascularization [72].

Additionally, IL-17A amplifies the angiogenic cascade by stimulating macrophages to secrete potent pro-angiogenic factors, including VEGF and IL-6, thereby further fueling corneal neovascularization and exacerbating vascular growth [73]. Evidence also suggests that TNF-α may potentiate angiogenesis by upregulating angiogenic mediators and promoting macrophage recruitment to the site of corneal alkali burn injury [74].

Our findings further reveal that RAPA treatment distinctly reduces VEGF expression by day 14 (Figure 7A), accompanied by a decrease in CD31^+^ expression, indicative of suppressed angiogenic activity. These findings align with prior reports showing that RAPA inhibits mTOR-dependent VEGF signaling and endothelial cell activation, thereby disrupting the inflammation-driven pathological feedback loop between angiogenesis and immune cell activation in corneal alkali burn injury models [21,75]. Collectively, these results support RAPA’s potential to effectively mitigate aberrant neovascularization through targeted modulation of VEGF and CD31-positive endothelial vessel formation by suppressing the upstream inflammatory triggers that sustain pathological vessel growth.

Critically, fibrotic remodeling following alkali burn injury underlies corneal haze and long-term visual impairment [1,76]. Persistent inflammation primarily drives this fibrotic response by promoting the differentiation of corneal fibroblasts into contractile myofibroblasts characterized by increased α-SMA expression. These myofibroblasts mediate excessive ECM deposition and stromal disorganization, leading to scar tissue formation and loss of corneal transparency (Figure 8A) [36,76,77]. MMPs, especially MMP-9, which are predominantly secreted by basal corneal epithelial cells, along with tissue inhibitors of metalloproteinase (TIMPs), exacerbate the fibrotic cycle by accelerating corneal basement membrane breakdown and excessive ECM degradation [78]. This fibrotic process, in turn, intensifies proinflammatory cytokine activity and promotes aberrant angiogenesis.

Supporting this, previous studies in a mouse model of corneal alkali burn injury demonstrated that IL-1β-mediated inflammation plays a central role in promoting fibrosis by enhancing α-SMA expression and myofibroblast differentiation [79,80]. These effects exacerbate tissue damage, impair corneal regeneration, and ultimately lead to fibrosis and scarring [81,82,83]. Moreover, MMP-9 activity facilitates the activation and differentiation of fibroblasts into myofibroblasts, further amplifying the fibrotic response [84].

In this context, the suppression of both α-SMA expression and MMP-9 activity by RAPA highlights its capability to interrupt these key initiators of corneal fibrosis [24,29]. By mitigating inflammation-driven matrix degradation and reducing myofibroblast persistence, RAPA may help preserve stromal transparency and integrity following severe corneal alkali burn injury, thus underscoring its promise as a restorative agent for reducing vision-impairing scarring during corneal wound repair.

In addition to the cellular alterations characterized in this study, corneal alkali burn injury disrupts stromal microstructural integrity, including collagen fibril organization and proteoglycan composition, which are critical for corneal transparency, stromal architecture, biomechanical stability, and wound healing. While these aspects were not directly investigated in the present work, a recent study demonstrated that in vivo administration of keratanase II enzyme can modulate corneal proteoglycan content in murine models, suggesting a potential complementary strategy to mitigate ECM alterations induced by alkali burn. Such enzyme-based interventions may help preserve stromal architecture, reduce fibrosis, and enhance overall corneal transparency, thereby representing a promising avenue for future therapeutic exploration [85,86].

Moreover, maintaining a stable tear film and an intact corneal epithelial surface is fundamental for preserving ocular surface homeostasis and restoring visual function following corneal alkali burn injury [23]. However, alkali-induced ocular surface damage can severely impair tear film stability by disrupting the corneal epithelium, conjunctival goblet cells, and lacrimal glands. This tear deficiency creates a pro-inflammatory microenvironment that worsens tissue damage, hinders epithelial regeneration, and ultimately delays wound healing [55].

Importantly, our study demonstrates that topical RAPA treatment noticeably improves corneal wound healing by promoting reepithelialization and restoring tear film stability. Specifically, RAPA-treated mice exhibited boosted tear production compared to injury-only mice, indicating functional recovery of secretory tissues alongside marked attenuation of local inflammation. Although RAPA’s ability to modulate inflammatory responses and prevent glandular dysfunction is well documented in other disease models [22,87], its direct impact on tear secretion following corneal alkali burn injury remains less explored. These beneficial outcomes are likely attributable to RAPA’s suppression of proinflammatory cytokine expression and its preservation of epithelial and goblet cell viability, which together support ocular surface homeostasis following corneal alkali burn injury [88,89].

ZO-1 is an integral tight junction protein that reflects corneal epithelial barrier function, which is severely disrupted following corneal alkali burn injury due to extensive epithelial damage and sustained inflammatory responses [42,90]. Our results show that RAPA treatment substantially restored continuous ZO-1 expression across the regenerating corneal epithelium, confirming enhanced barrier recovery (Figure 3A). Notably, this demonstrates a previously underexplored benefit of topical RAPA in corneal alkali burn injury, its ability to restore epithelial barrier integrity. This effect is closely tied to RAPA’s potent anti-inflammatory action, which reduces cytokine-driven epithelial disorganization and preserves epithelial sheet integrity by limiting inflammatory cell infiltration and dampening pro-inflammatory signaling. These findings correspond with previous results in a mouse model of corneal alkali burn injury, where increased TNF-α levels were shown to downregulate ZO-1 expression, directly linking inflammation to tight junction disruption [41]. As a result, RAPA supports corneal epithelial barrier stability and promotes tight junction reassembly, both vital for effective corneal wound healing.

A hallmark of corneal alkali burn injury is corneal epithelial disruption, evidenced by increased fluorescein staining and delayed wound closure, resulting from damage to the epithelial barrier and impaired reepithelialization as extensively documented in previous studies [36,38,91,92]. In our evaluation, we found that RAPA treatment sharply reduced fluorescein staining scores over time, validating enhanced epithelial closure and restoration of corneal barrier function. Similar to our findings, previous studies have demonstrated that RAPA favors epithelial repair in various ocular disease models, playing a critical role in regulating inflammation and epithelial cell proliferation [22,93]. This observed decrease in epithelial defect area and fluorescein retention in RAPA-treated eyes suggests that its immunomodulatory effects not only suppress inflammation but also facilitate faster epithelial regeneration, thereby contributing to improved corneal wound healing following corneal alkali burn injury.

A pivotal finding is that RAPA effectively downregulates both cellular apoptosis and proliferation, which are essential for balanced tissue repair following corneal alkali burn injury. Alkali burn injury typically causes excessive apoptotic cell death, reflecting the severity of tissue injury, alongside uncontrolled compensatory proliferation marked by Ki67^+^ expression. Together, these processes lead to disorganized healing and scar formation [30,36,42]. Our observation is in line with previous studies that demonstrate mTOR inhibition plays a key role in regulating cell cycle progression and enhancing cell survival [94]. Prior studies have shown that IL-1β induces corneal epithelial apoptosis, contributing to the imbalance in cell turnover during alkali burn injury [62,79]. Importantly, in the presence of topical RAPA, we show a coordinated modulation of apoptosis and proliferation, linking its anti-inflammatory effects with improved wound healing, which represents a mechanistic insight in the context of corneal alkali burn injury. By restoring the balance between cell death and hyperproliferative cellular activity, RAPA creates a more organized and functional repair environment, leading to improved structural integrity and better clinical outcomes. This combined effect underscores RAPA’s multi-target pleiotropic role in mitigating inflammation driven corneal damage while promoting effective regeneration.

Despite these promising results, several limitations should be considered. Our study utilized a mouse model of corneal alkali burn injury, which, while widely accepted and reproducible, may not fully capture the complexity of human corneal alkali burn injuries. Differences in corneal anatomy and immune response between species could affect the translational applicability of our findings [53]. Additionally, this study primarily focused on short to mid-term outcomes following RAPA treatment; long-term effects on corneal healing, visual acuity, and possible adverse effects remain to be investigated. Future studies with extended follow-up periods are needed to assess the sustainability and safety of RAPA therapy. Likewise, although RAPA demonstrated broad curative efficacy in mitigating corneal alkali burn injury, further investigation is warranted to delineate its downstream signaling mechanisms, cellular targets, toxicity, and prospective off-target effects to optimize its clinical application. Beyond this, the dosage and delivery method of RAPA in this study were selected based on previous research and may not reflect the optimal clinical regimen for human use. Alternative delivery strategies, such as sustained release systems, should be investigated to enhance efficacy and reduce systemic exposure. Future studies should compare RAPA’s efficacy with steroid eye drops to assess its promise as an adjunct therapy to reduce steroid use. Nevertheless, continued research should aim to optimize dosing regimens and explore combinatorial approaches to fully harness the regenerative capacity of RAPA in corneal alkali burn injury.

To the best of our knowledge, this study represents a milestone in integrating both injury-induced processes, including immune activation, inflammation, neovascularization, fibrosis, myofibroblast activation, ECM remodeling, apoptosis, and proliferation, and functional outcomes such as tear production, epithelial barrier integrity, and wound closure in a clinically relevant topical RAPA mouse model of corneal alkali burn injury. These findings demonstrate RAPA’s multi-target, pleiotropic therapeutic effects, with inflammation as the central driver linking these processes, establishing a strong rationale for its clinical exploration in corneal wound healing.

Together, these findings position RAPA as an innovative treatment modality capable of addressing the complex, inflammation-driven pathology of corneal alkali burn injuries. Further research exploring optimized delivery systems and long-term outcomes may prove decisive for advancing clinical translation.

## 5. Conclusions

In summary, our study demonstrates that topical RAPA promotes corneal wound healing by targeting multiple injury-induced pathological processes and improving functional outcomes following alkali burn injury. These findings position topical RAPA as a promising multi-target immunomodulatory paradigm with strong translational relevance. Overall, our work provides a foundation for inflammation-centered therapies aimed at comprehensive and sustained recovery in severe corneal alkali burn injuries.

## Data Availability

All datasets used and/or analyzed during the current study are available from the corresponding author on reasonable request.

## Abbreviations

RAPA	Rapamycin
ECM	Extracellular Matrix
VEGF	Vascular Endothelial Growth Factor
NSAIDs	Nonsteroidal Anti-Inflammatory Drugs
mTOR	Mammalian Target of Rapamycin
MMP-2	Matrix Metalloproteinase-2
bFGF	Basic Fibroblast Growth Factor
IL-1β	Interleukin 1-beta
TNF-α	Tumor Necrosis Factor-Alpha
IL-6	Interleukin 6
IL-17B	Interleukin-17B
CD31	Cluster of Differentiation 31
CD45	Cluster of Differentiation 45
MMP-9	Matrix Metalloproteinase-9
ZO-1	Zonula Occludens-1
α-SMA	Alpha-Smooth Muscle Actin
TUNEL	Terminal Deoxynucleotidyl Transferase UTP nick end labeling
NaOH	Sodium Hydroxide
TIMPs	Tissue Inhibitors of Metalloproteinases
TLR4	Toll-Like Receptor 4
NLRP3	NOD-Like Receptor Family Pyrin Domain Containing 3
H&E	Hematoxylin and Eosin
MT	Masson’s Trichrome
